# Senecavirus cetus a novel picornavirus isolated from cetaceans represents a major host switching to the marine environment

**DOI:** 10.1038/s44298-024-00040-6

**Published:** 2024-08-02

**Authors:** Oksana Vernygora, Daniel Sullivan, Ole Nielsen, Kathleen Burek Huntington, Natalie Rouse, Vsevolod L. Popov, Oliver Lung

**Affiliations:** 1 Canadian Food Inspection Agency, National Centre for Foreign Animal Disease, Winnipeg, MB Canada; 2 Department of Biological Sciences, University of Manitoba, Winnipeg, MB Canada; 3 Department of Fisheries & Oceans Canada, Winnipeg, MB Canada; 4 Department of Biological Sciences, University of Alaska Anchorage, Anchorage, AK USA; 5 Alaska Veterinary Pathology Services, Eagle River, AK USA; 6 Center for Biodefense and Emerging Infectious Diseases, University of Texas Medical Branch, Galveston, TX USA

**Keywords:** Genetics, Viral evolution, Virology

## Abstract

*Senecavirus A* (SVA), an emerging virus that causes vesicular disease in swine, was, until recently, the only member of the *Senecavirus* genus (*Picornaviridae*). Here, we report the isolation and complete genome sequence of two isolates of cetacean picornavirus 1 (*Senecavirus cetus*), a novel picornavirus species of the *Senecavirus* genus from dead stranded cetaceans from Alaska. One isolate was from a harbor porpoise stranded in 2017, and another from a beluga whale, stranded in 2019. Whole-genome sequencing of *Senecavirus cetus* strains showed a genome-wide nucleotide identity of 98.8% and a genome size of 7455 nucleotides. The *Senecavirus cetus* genomes are most similar to SVA with a 58.3% genome-wide pairwise nucleotide identity. Infection of eleven available cell lines from terrestrial and aquatic animals showed that beluga and sheep cells were susceptible to infection by *Senecavirus cetus*. Phylogenetic and ancestral state reconstruction analyses supported the novel virus being a member of the *Senecavirus* genus and provided the first evidence of *Senecavirus*-like picornavirus infecting marine mammals and likely descending from a terrestrial host ancestor. These discoveries provided important information on the evolutionary relationships and taxonomy of picornaviruses and increased our understanding of the genomic characteristics and potential host range of *Senecavirus cetus*.

## Introduction

*Picornaviridae* (Order *Picornavirales*) is a large, diverse family of small, non-enveloped, icosahedral RNA viruses with spherical 20–30 nm diameter nucleocapsids and a single-stranded, positive-sense RNA genome ranging between 6.7 and 10.1 kb in size^1^. The highly diverse genome consists of a distinguishing characteristic long 5′ UTR with an internal ribosomal entry site (IRES) 250–450 nucleotide upstream of the translational start site, a single ORF encoding a polyprotein, a shorter 3′ UTR, and a poly-A tail. However, the genome of Dicipiiviruses (genus *Dicipivirus*) has two ORFs, each with its own IRES. Based on its secondary structure, most picornavirus IRES elements are currently classified into five types (I-V, IGR-IRES). *Picornaviridae* is constantly adding new members due to advances in next-generation sequence technology and testing of clinical and environmental samples^2^ and is considered to include 68 genera and 158 species as of September 11, 2023, the date of the latest International Committee on the Taxonomy of Viruses (ICTV) master species list. Picornaviruses infect a wide range of hosts, including amphibians, reptiles, fish, birds, humans, and other mammalian species, including marine mammals. Infections are usually mild and subclinical, but more severe presentations include fever, diseases of the heart, nervous system, respiratory tract, gastrointestinal tract, liver, and vesicles on mucocutaneous tissues^2,3^. Some examples of human diseases include the common cold associated with rhinovirus A, B, and C^4^, poliomyelitis caused by poliovirus^5^, and hepatitis A caused by hepatovirus^6^. Picornaviruses are also the cause of notable veterinary diseases, including foot-and-mouth disease (FMD), which causes enormous economic losses in swine and cattle industries worldwide^7^, and Senecavirus-associated vesicular disease (SAVD) of swine, which presents as clinically identical to FMD in swine, necessitating confirmatory diagnostic testing^8^.

Senecavirus A (SVA) was first isolated in the human primary embryonic retinoblastoma cell line PER.C6 as a rapidly growing tissue culture contaminant in 2002, likely from a contaminated porcine trypsin reagent. SVA is capable of in vitro growth in several cell lines, including those of porcine and non-porcine origin, i.e., porcine kidney-15 (PK-15), swine testis (ST), human primary embryonic retinoblastoma cell line (PER.C6), and a human lung cancer cell line (NCI-H1299). Purified virus sample and virus-infected PER.C6 cells revealed icosahedral particles of about 27 nm in diameter, consistent with other members of the *Picornaviridae*. Subsequently, more isolates were isolated, including an isolate from a buffalo (*Bubalus bubalis*) from China^9^, and genome sequencing confirmed it as a novel monotypic genus within the family *Picornaviridae*, and most closely related to the genus *Cardiovirus*^10^. Among SVA’s other notable attributes are that it is incapable of integrating into human genomic DNA during infection and its ability to selectively infect and kill human tumor cells after some genetic modification, making SVA an ideal virus for developing oncolytic virus cancer therapies^11^.

It was not until 2007 that SVA was shown to be responsible for vesicular disease and lameness in a shipment of pigs from Manitoba, Canada^12^. The vesicles were indistinguishable from those caused by FMD, swine vesicular disease (SVD), vesicular stomatitis (VS), and vesicular exanthema (VE), but when vesicle material was tested, they were only positive for SVA, confirming its ability to cause these clinical manifestations. It was also associated with new clinical presentations, such as diarrheal disease in newborn piglets and neonatal mortality, suggesting it was becoming more pathogenic^13^. From 2007 to 2015, SVA was detected sporadically in pigs in the USA and Brazil. However, after 2015, SVA spread rapidly to Asia, the UK, as well as Mexico, Colombia, and Chile in the Americas, causing severe problems in sows and finishing pigs in China, in addition to neonatal mortality^14^. To date, many aspects of SVA’s biology, including its origin, natural reservoirs, and transmission pathways, remain unknown. However, the detection of SVA-neutralizing antibodies, SVA in mice’s small intestine, feces, and genomic material in flies from unaffected farms suggest they may play a role in its epidemiology^15^.

**Fig. 1:**
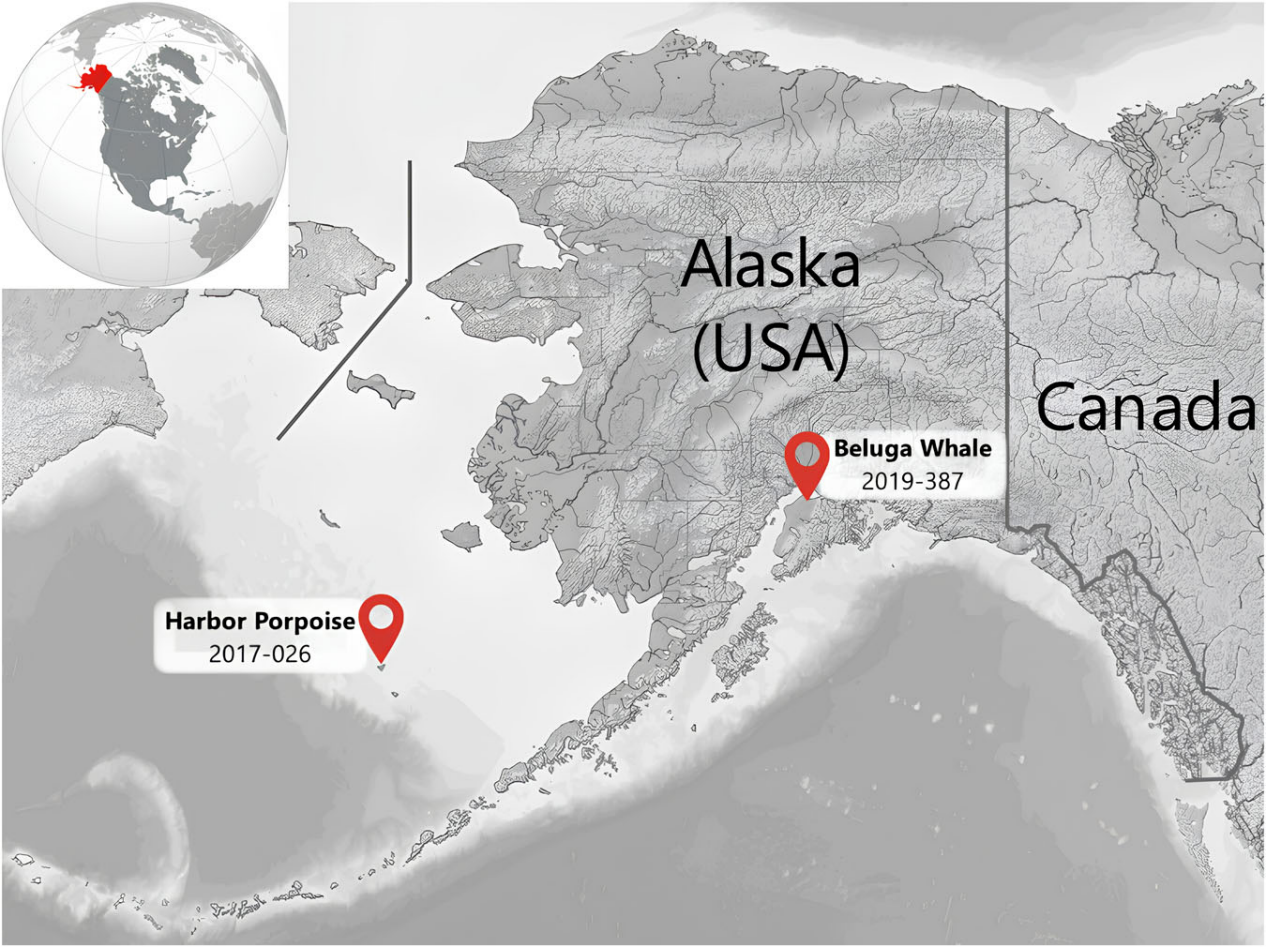
Map showing locations where the deceased animals were collected. A 4–5-year-old male harbor porpoise was found dead on St Paul Island, Alaska, USA on May 11, 2017 and a 1.5–2-year-old male beluga (*Delphinapterus leucas*) was found stranded dead, on September 2, 2019, at Campbell Creek, Turnagain Arm, Alaska.

Several picornaviruses have been characterized by phocids and a single cetacean. The majority of these viruses were described from metagenomic surveys, but some have been cultured, including harbor seal (*Phoca vitulina*) picornavirus (HsPV) and ribbon seal (*Histriophoca fasciata*) picornavirus (RsPV)^16^; ringed seal (*Phoca hispida)* picornavirus 1 (SePV-1)^17^. Whereas Megrivirus E1 isolate from Adelie penguin feces^18^, sub-Antarctic fur seal (*Arctocephalus tropicalis)* sakobuvirus, fur seal (*Arctocephalinae spp*.) picorna-like virus, South American fur seal (*Arctocephalus australis)* picornavirus^19^; harbor seal phopivirus, harbor seal Hepatovirus B^20^; California sea lion (*Zalophus californianus)* sapelovirus 1 and 2^21^, Fur seal picorna-like virus from *Arctocephalus gazelle*^22^ have not been isolated in cell culture. A single report of a picornavirus having been cultured from a cetacean, the bottlenose dolphin *Tursiops truncatus* (bottlenose dolphin enterovirus), was previously described^23^. The partial genome sequence of picornavirus from a captive beluga, HMU-1, has also been reported^24^. The role of marine mammal picornavirus infection in causing disease is presently unknown. SePV-1 was isolated from a nasal swab of a hunter-harvested, apparently healthy, ringed seal from arctic Canada^17^, while HsPV and RsPV were both isolated from dead stranded seals but what role the infection had in contributing to their death is unclear^16^. In this study, we report the first isolation of picornaviruses from harbor porpoise and beluga from Alaska (Fig. 1), sequenced the complete genomes, and performed phylogenetic/genetic analyses that demonstrated these cetacean picornaviruses represent a novel species.

## Results

### Pathology

#### Beluga whale 2019387

**Fig. 2:**
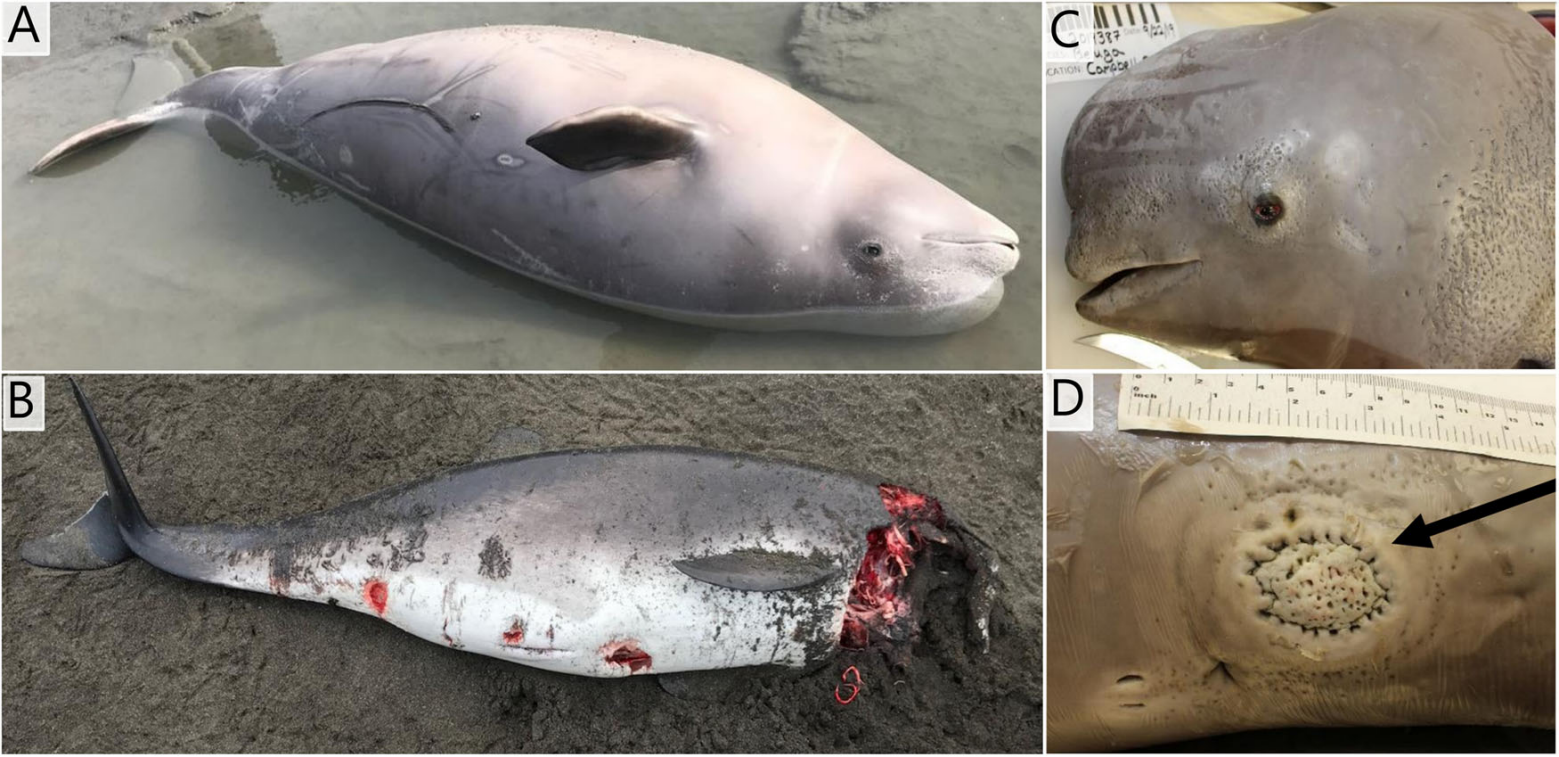
Carcasses of the sampled animals at the sites of discovery and close up images of some pathologies identified during necropsy. Beluga whale (*Delphinapterus leucas*) (**A**, **C**, **D**) and Harbor porpoise (*Phocoena phocoena*) (**B**). **A** is an overview of the beluga, **C** shows the small punctuate ulcers over the head and neck and the arrow in (**D**) points to a large, round lesion with a circular pattern of ulcers along the periphery. **B** is an overview of the harbor porpoise.

Grossly, this male subadult beluga had punctuated skin ulcers on the head and body, focal ulcerative and proliferative skin lesions (Fig. 2A, B), signs of possible blunt trauma to the right side body and head, an incompletely healed umbilicus, pulmonary lymphadenopathy, and pancreatitis.

Histopathology revealed moderate, chronic lymphoplasmacytic and eosinophilic dermatitis, with some lesions having intranuclear viral inclusion bodies suggestive of herpesvirus. There was also chronic, moderate to severe lymphohistiocytic multifocal pleuropneumonia—likely related to lungworms. The tonsil was reactive, and the thymus had acute mild multifocal random suppurative thymitis with lymphocytolysis and mild thymic atrophy. There was lymphocytolysis in the hilar lymph node, mild, multifocal degenerative myopathy, serous atrophy of fat, severe chronic ductal pancreatitis with intralesional trematode eggs, and mild eosinophilic (parasite-related) enteritis. The immediate cause of death was determined to be blunt trauma or possible live stranding, with chronic pleuropneumonia, poor body condition, dermatitis, and parasitism as contributing factors.

Ancillary diagnostics revealed the lung to be positive for *Erysipelothrix rhusiopathiae* (large numbers)*, Streptococcus dysgalactiae*, and *Vibrio sp*. (very small numbers) by culture and negative for morbillivirus and influenza virus by PCR. Heart and spleen were positive for *Erysipelothrix spp*. bacteria by PCR, suggesting this animal was also septic for *Erysipelothrix* sp. Skin lesions were negative for poxvirus and positive for beluga alphaherpesvirus^25^ (Fig. 2) by PCR and sequencing.

#### Harbor porpoise 2017026

Grossly, this male subadult harbor porpoise had trauma to the head, including a missing tongue and lower jaw, most consistent with killer whale predation (Fig. 2B). There was also poor body condition, severe pulmonary edema, bronchopneumonia due to nematodes and bacteria, chronic cholangiohepatitis and pancreatitis due to heavy trematode infection, and gastritis due to heavy nematode infection. The cause of death was determined to be predation of an animal in poor condition with pre-existing disease.

### Virus isolation and transmission electron microscopy

Harbor porpoise rectal swabs and liver samples inoculated onto BWK cells showed the development of granular CPE after 5 and 6 days post-infection (DPI), respectively. The beluga brain sample developed a similar CPE after 26 DPI. In all cases, CPE developed very quickly, with the cell monolayer being completely destroyed after an additional 48 h of incubation. None of the VeroDog.SLAMtag-infected flasks showed any signs of CPE after 4 weeks of incubation.

**Fig. 3:**
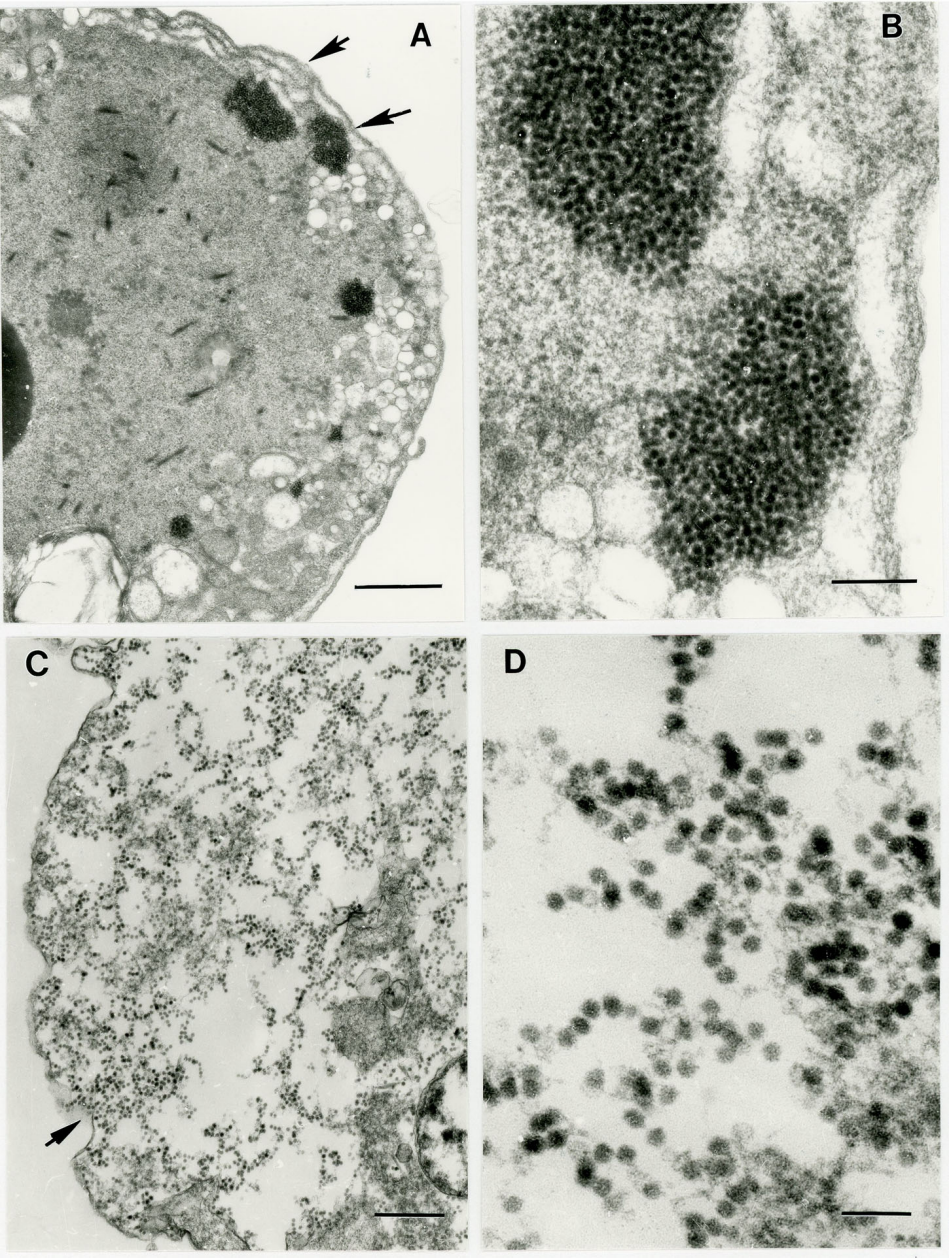
Ultrastructure of *Senecavirus cetus* isolate from harbor porpoise (2017026) in BWK cells. **A** Portion of a dense BWK cell infected with liver isolate showing cytoplasmic clusters of virus particles and multiple vesicles 130–270 nm in diameter at the cell periphery. Scale bar = 1 µm; **B** High magnification of two clusters indicated by arrows in (**A**) consisting of individual virus particles ~25 nm in diameter; **C** Portion of a BWK cell with lysed cytoplasm infected with a rectal swab isolate loaded with individual virus particles. Scale bar = 500 nm; **D** High magnification of an area indicated by an arrow in (**C**) showing individual round non-enveloped virus particles ~25 nm in diameter. Scale bar = 100 nm.

In ultrathin sections of BWK cells infected with liver isolate, dense cytoplasmic clusters of viral particles were observed (Fig. 3A). They consisted of individual non-enveloped virions ~25 nm in diameter (Fig. 3B). Usually, these areas contained multiple vesicles 130–270 nm in diameter typical for picornavirus replication sites. In BWK cells infected with rectal swab isolate virus particles were dispersed in the cytosol of completely lysed cells (Fig. 3C, D).

### Cell line susceptibility

CPE developed in all the infected flasks of the BWK cell line after 48 h and the BTEC cell line after 9 DPI. Both cell lines originated from beluga. The two cell lines derived from sheep (OA3.Ts and OA4.K/S1) also showed CPE after 9 DPI. None of the other cell lines tested showed visible signs of CPE after growth and passage for 1 month (Table 1).

**Table 1:** Animal cell line susceptibility results

	Dilution			
	1/10	1/100	1/1000	Uninfected control
BWK	cpe 48 h	cpe 48 h	cpe 48 h	Negative
BTEC	cpe 9 DPI	cpe 9 DPI	cpe 9 DPI	Negative
OA3.Ts	cpe 9 DPI	cpe 9 DPI	cpe 9 DPI	Negative
OA4.K/S1	cpe 9 DPI	cpe 9 DPI	cpe 9 DPI	Negative
Vero.DogSLAMtag	Negative	Negative	Negative	Negative
RSM	Negative	Negative	Negative	Negative
RSPK	Negative	Negative	Negative	Negative
CRFK	Negative	Negative	Negative	Negative
PK-15	Negative	Negative	Negative	Negative
ST	Negative	Negative	Negative	Negative
MDBK	Negative	Negative	Negative	Negative

Results marked as “cpe” (cytopathic effect) indicate complete destruction of the cell sheet at the indicated time point (DPI—days post infection); results marked as negative indicate no visible signs of cytopathic effect at the end time point of the testing.

### Genome sequencing

#### Whole genome sequencing

**Fig. 4:**
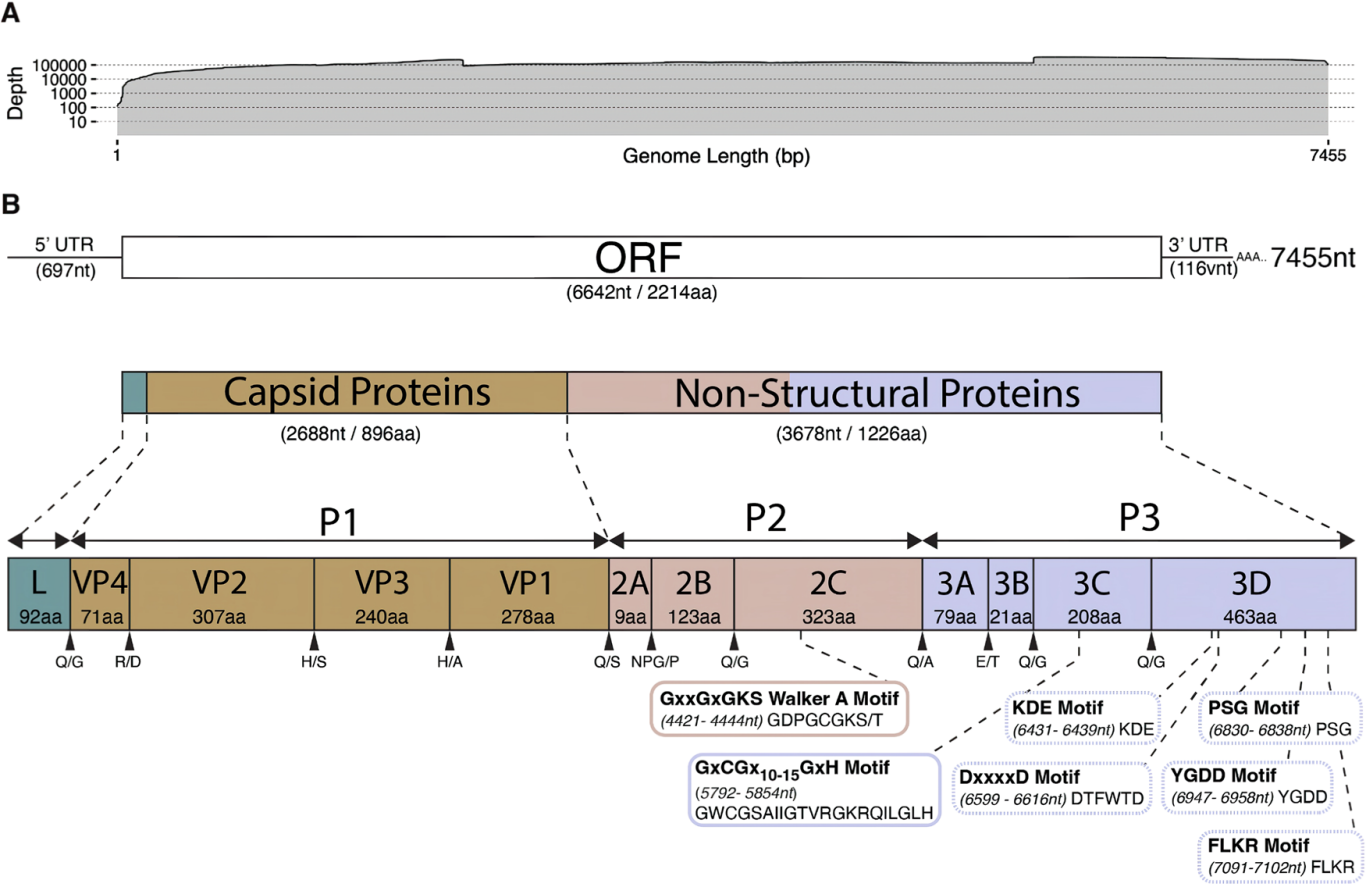
*Senecavirus cetus* genome information. **A** Total breadth and depth of coverage across the *Senecavirus cetus* genome using combined 5’/3’ RACE, Oxford Nanopore Rapid PCR Barcoding and Illumina Virocap data. **B** Predicted *Senecavirus cetus* genome layout and organization. The ORF1 (6624nt/2214aa) and flanking 5’ (697nt) and 3’ (116nt) regions can be found at the top. In the middle; the ORF1 is broken down into the capsid (2688nt/896aa) and non-structural proteins (3678nt/1226aa). A leader (L—92aa) protein is found before the P1 region containing the structural/capsid proteins VP1–VP4 (2688nt/896aa). The P2 and P3 regions represent the non-structural proteins 2A–2C and 3A–3D (3678nt/1226aa). Protein names along with amino acid lengths are found within each segment. The predicted protein cleavage sites are indicated in between each gene segment at the bottom.

The whole genome of the *Senecavirus cetus* (CPV1, beluga brain) isolated from the brain of a stranded dead beluga (*Delphinapterus leucas*) from Alaska, USA, in 2019 was 7455 nt in length. The genome organization of CPV1 and the depth of coverage are shown in Fig. 4. A total of 1.79 million reads were mapped across the CPV1 genome with a mean depth of coverage of 167,903x. CPV1’s genome has a 697 nt 5′UTR, followed by a 6642 nt that encodes a 2214 aa polyprotein and a 116 nt 3’UTR. The polyprotein has a typical 4-3-4 arrangement that codes for the structural proteins followed by non-structural proteins. Comparative genomic analysis of the complete coding genome for CPV1 found that conserved motifs present within members of the *Picornaviridae* family were present, including the Walker A motif (GxxGxGKS/T), 3C proteinase motif (GxCGx10–15GxH) and 3D polymerase motifs (KDE, DxxxxD, PSG, YGDD, FLKR).

**Fig. 5:**
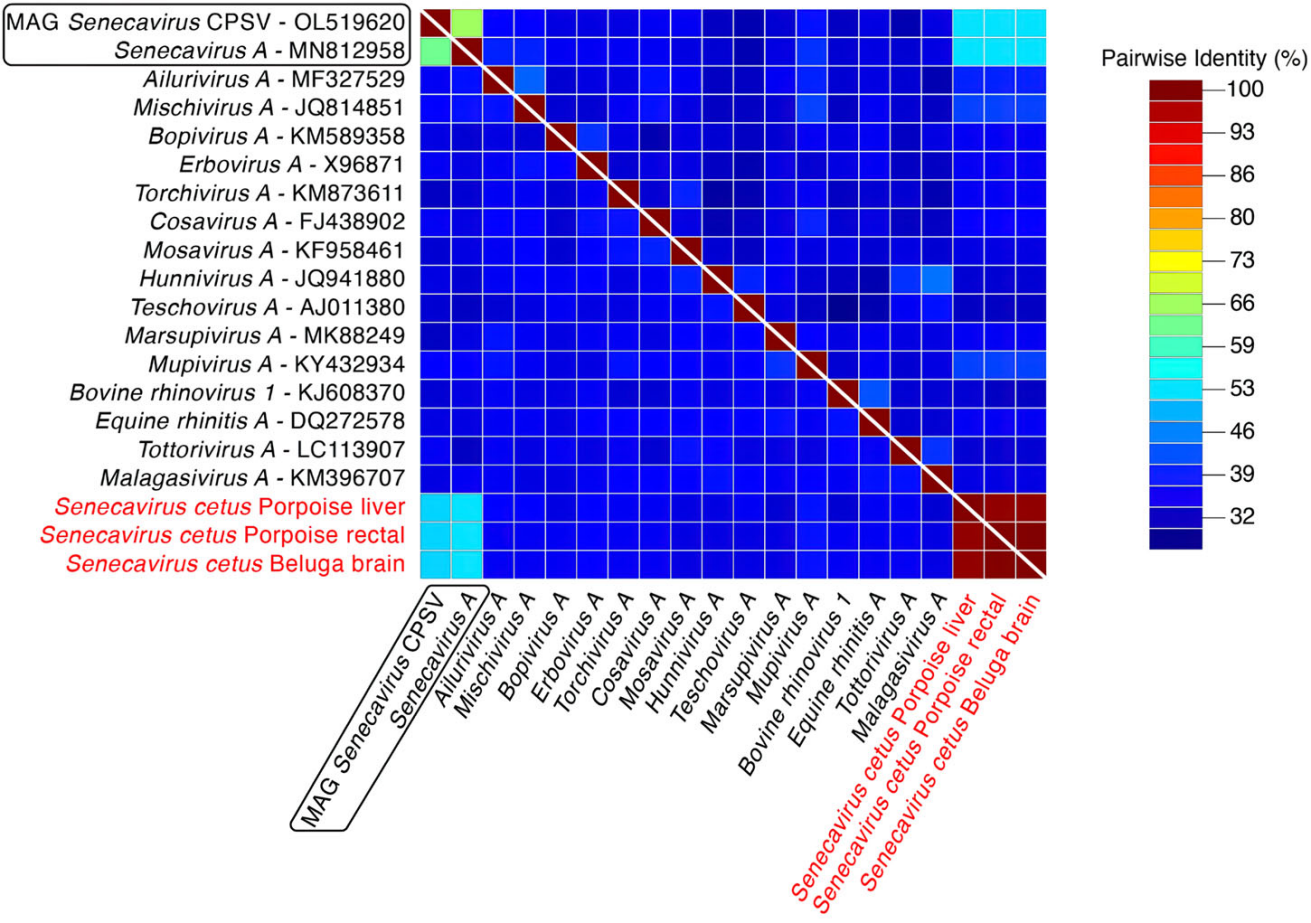
Pairwise identity of *Senecavirus cetus* and closely related viruses. ORF1 pairwise nucleotide (bottom left) and amino acid (top right) identity matrix for novel CPV1 isolated in beluga (*D. leucas*) and porpoise (*P. phocoena*) compared to other picornaviruses from the subfamily Caphthovirinae. A diagonal white line is used to separate both halves of the figure. Novel CPV1 isolates are highlighted in red while *Senecavirus A* (MN812958) and Senecavirus from pangolin (OL519620) are circled with a black box as other members within the *Senecavirus* genus.

**Fig. 6:**
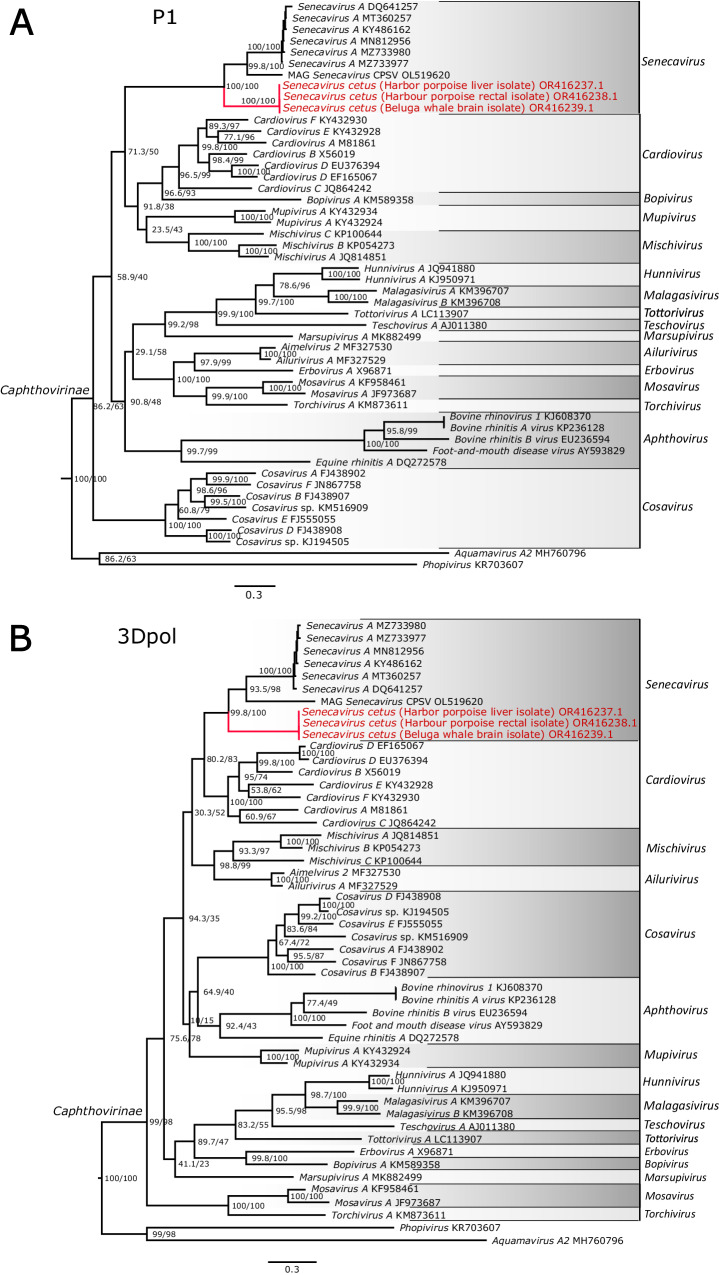
Phylogenetic relationships of picornaviruses in the subfamily Caphthovirinae. Maximum likelihood phylogenetic trees of picornaviruses in the subfamily Caphthovirinae reconstructed based on the nucleotide sequences of the P1 (**A**) and 3Dpol (**B**) genes. Sequences of the novel picornavirus are shown in red; the most closely related lineage, genus Senecavirus, includes a Senecavirus identified in pangolin, OL519620 MAG Senecavirus CPSV^35^. Phylogenetic analysis was performed using IQ-Tree web server 1.6.12. The trees were rooted to the outgroups, *Phopivirus* and *Aquamavirus A2*, member of the subfamilies Heptrevirinae and Paavivirinae, respectively. Scale bars represent estimated average number of substitutions per site.

BLAST analysis of the CPV1 sequence shows Seneca Valley virus (species Senecavirus A, SVA isolate USA/MI16-038766/2016 polyprotein gene, accession MN812958.1) as the top match. CPV1 and the SVA top match showed an ORF1 pairwise nucleotide identity of 58.29% and an ORF1 amino acid identity of 51.32% (Table 2). Additionally, the genomes shared approximately 40.5% and 44.5% nucleotide identity for the 3Dpol and P1 protein-coding regions, respectively. The nucleotide and amino acid identity, and phylogenetic analysis (Figs. 5 and 6), suggest CPV1 should be classified as a new member within the *Senecavirus* genus.

**Table 2:** Comparison of *Senecavirus cetus* (beluga brain) polyprotein coding region to closest BLAST matches

Species name	GenBank accession	Percent Identity	
		Nucleotide	Amino acid
*Senecavirus cetus*	OR416239	–	–
*Senecavirus A*	MN812958	58.3	51.8
Pangolin senecavirus	OL519620	53.7	49.7

Two isolates very similar to the novel CPV1 were also isolated and sequenced from a stranded dead harbor porpoise (*Phocoena phocoena*) (Table 3). The CPV1 (porpoise rectal) isolate showed an ORF1 pairwise nucleotide identity of 100% and an ORF1 amino acid identity of 100% to CPV1 (beluga brain). In comparison, the CPV1 (porpoise liver) isolate showed an ORF1 pairwise nucleotide identity of 98.8% and an ORF1 amino acid identity of 99.0% to CPV1 (beluga brain) (Fig. 5).

**Table 3:** Summary of the three *Senecavirus cetus* isolates described in this study

Species name/acronym	Isolate ID	Country of Isolate	Host/isolation source	GenBank accession
*Senecavirus cetus/CPV1*	Alaska/2019	USA	*Delphinapterus leucas* (Beluga whale)/Brain	OR416239
	Alaska/2017	USA	*Phocoena phocoena* (harbor porpoise)/Liver	OR416237
	Alaska/2017	USA	*Phocoena phocoena* (harbor porpoise)/Rectum	OR416238

#### Confirmatory sequencing of infected cell lines

Sequencing of the infected cell lines confirmed the presence of *Senecavirus cetus* in the cell cultures showing CPE. Quality-checked and filtered reads obtained from the confirmatory sequencing were assembled to the reference genomes of the original *Senecavirus cetus* isolates using nf-virontus v.1.1.0^26^. Assembled genomes showed 100% nucleotide identity to the beluga brain (OR416239) and harbor porpoise rectal (OR416238) isolates.

### Phylogenetic and genetic analyses

**Fig. 7:**
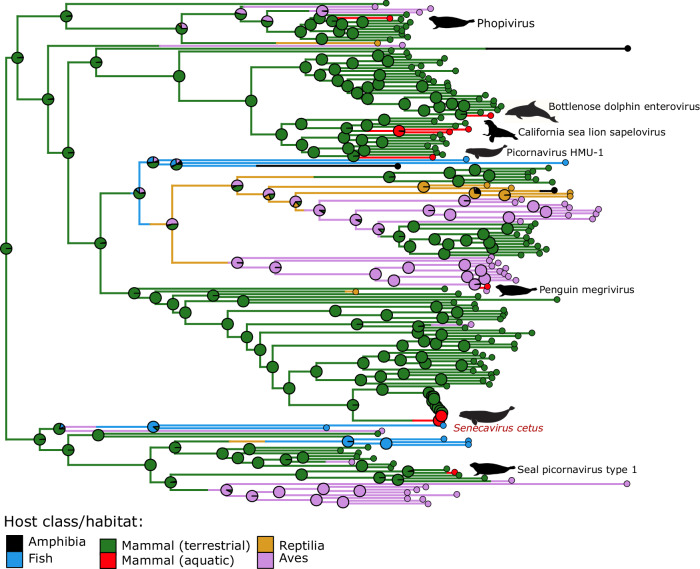
Ancestral state reconstruction of the host class and habitat within the family Picornaviridae based on the representative records of all recognized species. Tree is rooted at the midpoint. Pie charts at the nodes indicate the probability of each of the states at the ancestral nodes. Branches are coloured according to one of the 1000 replicates of the stochastic character mapping.

Phylogenetic analyses recovered *Senecavirus cetus* isolates forming a well-supported monophyletic group placed as a sister to a clade comprising a monophyletic SVA and a recently described member of the *Senecavirus* genus isolated from pangolin. This placement was consistent across the results of the whole genome, P1, and 3Dpol data set analyses (Fig. 6; Supplemental Fig. 1). *Mischivirus* and *Cardiovirus* were the two most closely related picornavirus lineages to the clade comprising SVA and *Senecavirus cetus*. Overall, picornaviruses isolated from marine mammals show broad phylogenetic distribution (Fig. 7; Supplemental Fig. 1) across multiple picornavirus subfamilies. Recombination analysis of the whole genome data set (*n* = 180 taxa) showed no evidence of recombination events in the genome of *Senecavirus cetus* and in neither of the other picornaviruses isolated from the aquatic hosts (Table 4). The ancestral state reconstruction suggested a terrestrial host habitat as the most likely state at the root of the *Picornaviridae* tree (Fig. 7). The inferred terrestrial origin of picornaviruses could, in part, be due to the fact that the majority of the diversity of this group of viruses has been described from terrestrial mammals representing a sampling bias. The most likely origin of the picornaviruses isolated from aquatic mammals was attributed to terrestrial mammals in nearly all cases, including the novel *Senecavirus cetus* reported herein. Additionally, a potential transition from an avian host to an aquatic mammal was identified in the case of Penguin megrivirus isolated from Weddell seal feces^18^.

**Table 4:** Picornaviruses isolated and/or directly sequenced from aquatic and semi-aquatic mammal hosts

Virus	Genus	Hosts	Isolate or direct sequencing	Reference
*Senecavirus cetus*	*Senecavirus*	Beluga whale (*Delphinapterus leucas*), harbor porpoise (*Phocoena phocoena*)	Isolate	This study
Phopivirus	*Hepatovirus*	Harbor seal (*Phoca vitulina*)	Sequencing	^20^
Fur seal picorna-like virus	unclassified	Antarctic fur seal (*Arctocephalus gazella*)	Not specified	Na (direct submission; KY926885.1)
Seal picornavirus type 1	*Aquamavirus*	Ringed seal (*Pusa hispida*)	Isolate	^17^
Penguin megrivirus	*Megrivirus*	Weddell seal (*Leptonychotes weddellii*)	Not specified	Na (direct submission; MN453782.1)
Picornavirus HMU-1	unclassified	Beluga whale (*Delphinapterus leucas*)	Sequenced	^24^
Bottlenose dolphin enterovirus	*Enterovirus*	Bottlenose dolphin (*Tursiops truncatus*)	Isolate	^23^
California sea lion sapelovirus 1	*Sapelovirus*	California sea lion (*Zalophus californianus*)	Sequenced	^21^
California sea lion sapelovirus 2	*Sapelovirus*	California sea lion (*Zalophus californianus*)	Sequenced	^21^

## Discussion

This paper describes the isolation and genomic sequences of two strains of a novel cetacean picornavirus, *Senecavirus cetus*. The two strains were isolated from a stranded dead beluga and a harbor porpoise in Alaska, USA, but two years and 1000 km apart. The genomic sequences of the two strains share 98.8% pairwise nucleotide identity and show the highest nucleotide identity (~53%) with SVA. SVA is an emerging swine virus that has been spreading and causing vesicular disease in pig populations in North America since its first detection in the 1980s and is now reported in the Americas, Asia, and the UK. Therefore, discovering a new *Senecavirus* species in marine mammals from Alaska highlights potential risks for animal health and food security in the region. Specifically, both beluga whales and harbor porpoises are preyed on by other marine predators such as orcas^27^, seals^28^, and sharks^29^, as well as by polar bears, a keystone Arctic species known to hunt and scavenge belugas^30–32^. Additionally, cetaceans, including belugas and porpoises, serve as an important food source for indigenous people^33,34^. The potential risks and impacts of the novel virus described in the present study remain to be investigated, but this work provides initial insight that may begin to address these concerns. Interestingly, the beluga had vesicular lesions throughout the carcass, resembling those caused by SVA in pigs. In addition to being found in pigs and the cetaceans reported in this study, a partial genome of a Seneca-like virus was recovered from a metagenomics study of the lung tissue of pangolin, family Manidae of the order Pholidota^35^, indicating that Seneca-like viruses may have a broader host range among terrestrial species.

The factors contributing to the emergence and evolution of the novel cetacean picornavirus are unknown. However, geographical, ecological, and other factors can contribute to spill-over events and drive virus evolution. Recombination and mutations are driving factors for the great diversity of picornaviruses. RNA recombination is observed in all studied picornavirus genera^2^. Phylogenetic analysis using the P1 and 3Dpol genomic regions shows *Senecavirus cetus* clusters with *Senecavirus A*. Mishivirus, with bats as natural hosts, and Cardiovirus, found in humans, were the two most closely related lineages to the clade comprising SVA and *Senecavirus cetus*. Overall, picornaviruses isolated from marine mammals show broad phylogenetic distribution, suggesting independent introductions and evolution in marine mammals. The ancestral state reconstruction shown here indicated that the origin of the picornaviruses in aquatic mammals traces back to terrestrial ancestors and, in a single case, potentially to an avian host (Fig. 7). The latter is most likely to be the case of horizontal transmission between host species as Weddell seals share habitat and feed on penguins in Antarctica^36,37^. Among the terrestrial to aquatic host transition cases, diverse phylogenetic patterns were observed where aquatic picornaviruses were inferred to descend from and be closely related to picornaviruses isolated from hosts across multiple deeply divergent lineages of mammals. *Phopivirus*, a distinct picornavirus species isolated from harbor seals, was inferred to share the most recent common ancestry with picornaviruses of small insectivore mammals such as hedgehogs and shrews. Together with other closely related *Hepatovirus* species of bats and rodents, these viruses form a group that includes the human hepatitis A virus. Another picornavirus species isolated from harbor and ribbon seals are co-generic and most closely related to the bear picornavirus described from an Asian black bear^38^. Similarly, aquatic picornaviruses from the subfamily Ensavirinae were found to be closely related to picornaviruses from phylogenetically diverse mammal hosts. PicoV-HMU-1 is a novel picornavirus discovered recently through metagenomics sequencing of swabs from captive belugas in an Ocean Park in China^24^. The PicoV-HMU-1 genome is most closely related to an unclassified bat picornavirus. Such a remarkably broad host range across diverse lineages of animals could indicate that the origin of the currently known picornaviruses predates the radiation of vertebrates. This is consistent with the previously proposed idea of the ancient origin of picorna-like viruses that predate the radiation of eukaryotes^39^ and an even earlier origin of their replication apparatus tracing back to the pre-cellular world^40^. However, tracing the origin of picornaviruses in aquatic hosts is greatly complicated by the sampling challenges associated with this type of habitat. This could also be a potential reason why no recombination events were detected in genomes of picornaviruses isolated from marine hosts since recombination detection approaches rely on the presence of the recombination donors (or closely related sequences) in a data set for recombination events to be identified.

To date, picornaviruses have been reported in five of seven vertebrate classes. However, genetic factors that influence the host range and pathogenicity for fish, avian, mammalian, and other picornaviruses are poorly understood. Due to the lack of data, the evolutionary history of aquatic mammalian picornaviruses will benefit from further knowledge of unsampled viral diversity. The genome sequences reported here will allow the development of diagnostic tests that will facilitate the investigation of archived, surveillance, and opportunistic samples and provide insight into the prevalence of picornaviruses in the host populations and the environment that can further shed light on the evolutionary mechanisms of picornaviruses. Many picornaviruses have been identified in the stools of animals and are known to be environmentally stable, suggesting this may be a plausible mechanism for cross-species spread. Likely scenarios for host switching include (1) between sympatric species that inhabit terrestrial and marine environments; (2) marine species that come in contact with and thus become infected through fecal contamination of environmental sources; (3) carnivores or scavengers infected through the diet after consuming infected animals. A prominent example of host switching in another family of the Picornavirales (*Caliciviridae*) is the transmission of San Miguel Sea Lion (vesicular exanthema) virus to domestic pigs that were fed sea lion meat at farms in the United States^41^. In swine hosts, the virus caused swine vesicular disease clinically similar to the Foot-and-Mouth disease caused by a picornavirus within the *Aphthovirus* genus of *Picornaviridae*. Cell line susceptibility studies revealed that only ones derived from beluga (BWK and BTEC) and sheep (OA3.Ts and OA4.K/S1) were susceptible to infection by *Senecavirus cetus*. In contrast, SVA causes CPE in a variety of mammalian and livestock cell lines, including the swine cell lines ST and PK-15 and the bovine cell line MDBK^42^, but these cell lines were unable to support the growth of the *Senecavirus cetus*. These results may indicate a further adaptation to the marine environment.

Unlike PicoV-HMU-1 and other picornaviruses with only sequences available, we report the first *Senecavirus cetus* isolates. The virus isolates can facilitate biochemical and molecular investigations into the functional analysis of transcription and translational regulatory sites, encoded proteins, precise poly-protein processing sites, host-virus interactions, host range, and zoonotic potential of *Senecavirus cetus*.

## Methods

### Animals and sample collection

A 4–5-year-old male harbor porpoise (NMFS ID#2017026) was found dead on St Paul Island, Alaska, USA, on May 11, 2017, in a moderate state of decomposition (Figs. 1 and 2). It was frozen and subsequently necropsied using standard techniques^43^. Viral swab samples (rectal) were placed in viral transport media (Remel, Lenexa, KS) and frozen at −80 °C. Tissue samples (adrenal, liver, and spleen) were frozen in Whirl-pak bags at −80 °C.

A 1.5–2-year-old male beluga (*Delphinapterus leucas*) (NMFS ID #2019387) was found stranded dead, on September 2, 2019, at Campbell Creek, Turnagain Arm, Alaska (Figs. 1 and 2) in a mild state of autolysis and was necropsied the following day using standard techniques^43^. Swabs and tissue samples (blowhole, rectal, genital, brain, lung, tonsil, pancreas, spleen, and skin lesions) were placed in viral transport media (Remel, Lenexa, KS) and frozen at −80 °C.

### Virus isolation and transmission electron microscopy

Frozen tissue and swab samples from both animals were processed for virus isolation using a beluga kidney cell line (BWK) and the VeroDog.SLAMtag cell line, both of which have been successful in isolating viruses from belugas^25^ and harbor porpoises^44,45^. Flasks were incubated at 37 °C and examined daily for cytopathic effects (CPE). Cells were passaged weekly (1:2) for BWK and (1:5) for VeroDog.SLAMtag for 4 weeks, at which time flasks not showing visible CPE were discarded. Media from flasks showing CPE was passed through a 0.45 μm filter, diluted (1/100), and passaged onto fresh cells. After CPE was again observed, the presumed virus-infected BWK flasks inoculated with the harbor porpoise liver and rectal swab presumptive isolates were processed for TEM as previously described^44^.

### Cell line susceptibility

Eleven cell lines derived from various species, tissues, and organs were used to investigate the host range of the beluga brain isolate (Table 5). In total, 25 cm^2^ flasks of each cell line were grown to confluence before being inoculated in duplicate with a 1/10, 1/100, or 1/1000 dilution of the virus passaged in BWK cells. After the virus was allowed to attach to the cells for 1 h at 37 °C, inoculum was removed, and fresh media was added before being returned to the incubator. Flasks were examined microscopically daily for signs of CPE and passaged weekly for up to a month. Flasks showing CPE were frozen at −80 °C and subsequently tested by sequencing to confirm the presence of *Senecavirus cetus*.

**Table 5:** Animal cell lines used in this study

Cell line	Host	Tissue	Continuous or Primary (Finite)	Source	Media^*^
BWK	Beluga (*Delphinapterus leucas*)	Kidney	Primary (Finite)	Developed in House	DMEM/F-12 + 10% FBS
BTEC	Beluga (*Delphinapterus leucas*)	Tracheal epithelium	Continuous	Developed in House	DMEM/F-12 + 4% FBS
OA3.Ts	Ovine (*Ovis aries*)	Testes	Continuous	ATCC CRL-6546	DMEM + 10% FBS
OA4.K/S1	Ovine (*Ovis aries*)	Kidney	Continuous	ATCC CRL-6549	DMEM + 10% FBS
Vero.DogSLAMtag	African green monkey (*Chlorocebus sabaeus*)	Kidney	Continuous	Dr. Yasuke Yanagi, Department of Virology, Kyushu University, Fukuoka, Japan	DMEM/F-12 + 4% FBS
RSM	Ringed seal (*Pusa hispida*)	Macrophage	Continuous	Developed in House	DMEM/F-12 + 4% FBS
RSPK	Ringed seal (*Pusa hispida*)	Kidney	Primary (Finite)	Developed in House	DMEM/F-12 + 10% FBS
CRFK	Domestic cat (*Felis catus*)	Kidney	Continuous	ATCC CCL-94	DMEM/F-12 + 4% FBS
PK-15	Swine (*Sus scrofa*)	Kidney	Continuous	ATCC CCL-33	AMEM + 5% Horse Serum
ST	Swine (*Sus scrofa*)	Testes	Continuous	ATCC CRL-1746	AMEM + 5% FBS
MDBK	Bovine (*Bos taurus*)	Kidney	Continuous	ATCC CCL-22	MEM + 5% Horse Serum

^*^Media formulation abbreviation: *AMEM* alpha modification of Eagle’s media, *MEM* minimal essential medium, *DMEM/F-12* Dulbecco’s modified Eagle medium/nutrient mixture F-12.

### Genome sequencing

#### Whole genome sequencing

Infected 75 cm^2^ flasks of BWK displaying extensive CPE from the beluga and harbor porpoise isolates were frozen and submitted to the National Centre for Foreign Animal Disease (NCFAD), Canadian Food Inspection Agency, Winnipeg, MB, Canada.

Nucleic acid extraction was performed using a combination of the Direct-zol™ RNA Miniprep Plus Kit and Quick-DNA/RNA™ Viral Kit (Zymo Research, Irvine, CA), including an on-column DNase I treatment according to the manufacturer’s instructions. First-strand cDNA synthesis was done using the SuperScript™ IV First-Strand Synthesis System (Invitrogen, Waltham, MA) with the inclusion of Endoh pseudorandom hexamers^46^, while second-strand synthesis was done using the NEBNext® Ultra II Non-Directional RNA Second Strand Synthesis Module (New England Biolabs, Ipswich, MA) according to the manufacturer’s instructions.

High-throughput sequencing was conducted using a combination of short and long-read sequencing technologies. For short-read sequencing, library preparation and enrichment were done following the ViroCap pan-vertebrate viral targeted enrichment method as previously described^47–49^ following the Nimblegen SeqCap EZ HyperCap Workflow User’s Guide (Roche, Basel, Switzerland). The resulting library was sequenced on an Illumina MiSeq using a V3 flow cell and 600-cycle kit (Illumina, San Diego, CA). For long-read sequencing, library preparation was done using the Rapid PCR Barcoding Kit—SQK-RPB004 (Oxford Nanopore Technologies, Oxford, United Kingdom) according to the manufacturer’s instructions, and the resulting library was sequenced on an Oxford Nanopore Technologies GridION using an SpotON Flow Cell—FLO-MIN106D (Oxford Nanopore Technologies, Oxford, United Kingdom).

Rapid amplification of cDNA ends (RACE) was used to capture the 5′ and 3′ ends of the genomes with the SMARTer® RACE 5′/3′Kit following the manufacturer’s protocol (Takara Bio, Mountain View, CA). Genome specific primers were designed approximately 1.5 kb from the assembled ends of the coding region using Primer3 (v0.4.0)^50^. The RACE primer sequences and characteristics are listed in Table 6. The amplified 5′ and 3′ RACE products were sequenced using the Native Barcoding sequencing kit—SQK-NBD114.96 (Oxford Nanopore Technologies, Oxford, United Kingdom) according to the manufacturer’s instructions and sequenced as described above using a SpotON Flow Cell—FLO-MIN114.

**Table 6:** 5′/3′ RACE primer information

Primer name	Sequence	Tm (°C)	Product size
*CPV1_5’_RACE*	CGCGGTTGTGTGGGAATAGGGCTG	68	2.1 kb
*CPV1_3’_RACE*	CTTCCCCTCCGCAACAGCCCCATC	69	1.7 kb

#### Confirmatory sequencing of infected cell lines

Presence of *Senecavirus cetus* in the infected cell lines displaying CPE from the susceptibility testing was confirmed using long-read high-throughput sequencing. Nucleic acid extraction and cDNA synthesis were performed following the same procedures as described above. Library preparation was performed using the Native Barcoding Kit—SQK-NBD114.96 (Oxford Nanopore Technologies, Oxford, United Kingdom) according to the manufacturer’s instructions. The final resulting libraries were sequenced on an Oxford Nanopore Technologies PromethION 24 sequencing platform using a PromethION FLO-PRO114M flow cell. An adaptive sampling strategy was used to deplete off-target reads during sequencing runs; reference genomes of domestic sheep (*Ovis aries*; RefSeq: GCF_016772045.2) and beluga whale (*Delphinapterus leucas*; RefSeq: GCF_002288925.2) were used as references for host depletion.

### Phylogenetic and genetic analyses

#### Raw data processing and genome organization

Raw Illumina sequence data was initially processed utilizing an in-house developed, automated classification pipeline; nf-villumina (v2.0.1)^51^ as previously described^47,52^. Raw Nanopore sequencing data was first basecalled using Guppy (v6.4.6) and super-accurate basecalling with a minimum q score cutoff of 10. 5’/3’ RACE Kit 14 Nanopore data was further processed with Duplex Tools (v0.3.2)^53^ to identify duplex read candidates prior to re-basecalling with Guppy (v6.4.6). Following, the basecalled data was subject to adapter trimming using Porechop (v0.2.4)^54^ and read length filtering with the chopper (v0.5.0)^55^ before consensus sequence generation using an in-house developed pipeline, nf-virontus (v1.0.1)^26^. Assembled contigs from both Illumina and Nanopore were further processed and analyzed in Geneious Prime (v2023.2.1) for both iterative mapping on medium-low sensitivity and 5 iterations and sequence alignment using MAFFT (v7.309)^56^. The resulting 5′/3′ RACE contigs and CPV1 contigs were checked for sequence overlap, and the final consensus was manually edited to generate the full-length genome. A combination of ORF finder, Protein BLAST, and MAFFT alignments were used to annotate the CPV1 genome. The total depth of coverage (Fig. 4A) was calculated by mapping both Illumina and Nanopore sequencing data against the complete CPV1 genome in Geneious Prime (v2023.2.1).

#### Pairwise identity analysis

Pairwise identity calculations for select members from the subfamily Caphthovirinae were done using the Sequence Demarcation Tool Version 1.2 (SDTv1.2)^57^. ORF1 nucleotide and amino acid sequences were aligned using the default MAFFT settings in SDTv1.2 while not selecting the “Cluster sequences using a neighbor-joining tree” option. A custom scale range of 29–100% pairwise identity was specified.

#### Phylogenetic analysis

The phylogenetic position of the novel cetacean picornavirus was assessed using the whole genomes and the P1 and 3Dpol gene sequences, two genomic regions conventionally used in the classification and phylogenetics of *Picornaviridae*. To construct the whole genome phylogenetic dataset, we used full genome data of all recognized genera of the family *Picornaviridae* (as of March 2023) and supplemented that data with available sequences of unclassified picornaviruses isolated from marine mammals (accessed on March 05, 2023). To assess the phylogenetic placement of the novel cetacean picornavirus relative to the closest species identified by the BLAST search, SVA, we included a total of ten SVA full genome sequences representing virus isolates from various geographical locations as well as the partial genome of a recently described novel picornavirus member of the *Senecavirus* genus isolated from pangolin. The final phylogenetic data set included 180 sequences. A more refined analysis was performed using the P1 and 3Dpol nucleotide sequences for all recognized genera within the subfamily Caphthovirinae, including SVA and the novel cetacean picornavirus. Details of the sequence records downloaded from GenBank and used in the study are provided in Supplementary Table 1. Multiple sequence alignment was performed using ClustalW^58^ v.1.8 implemented in BioEdit v.7.2.5^59,60^ under the default settings.

Phylogenetic analysis was performed using maximum likelihood implemented in IQ-Tree web server 1.6.12^61,62^. The best-fit model of sequence evolution was selected based on the Bayesian information criterion (BIC) score^63^ calculated by ModelFinder^64^, which recovered GTR + F + I + G4 as the best-fit model for both P1 and 3Dpol data sets. Maximum likelihood analyses were performed under the default settings with all model parameters estimated by the program. Node support was estimated by ultrafast bootstrap^65^ and the SH-aLRT test^66^ with 1,000 replicates each.

#### Recombination analysis

Genome recombination has been reported in a wide range of picornaviruses. To assess potential genome recombination in *Senecavirus cetus*, we performed a series of recombination analyses using the RDP5 v.5.30 software^67^ under the default settings and with the *p*-value cut-off set to 0.01. Five recombination detection methods (RDP, GENECONV, 3Seq, MaxChi, Chimaera) were used for the initial identification of putative recombination events in the whole genome sequence alignment. The identified recombination events were then checked with BootScan. Recombination events that were detected by all methods were selected as plausible.

#### Ancestral state reconstruction

The ancestral state reconstruction analysis was performed for the host habitat type to evaluate the most likely routes of evolutionary transitions between aquatic and terrestrial hosts. Character mapping and state reconstruction were performed in R using *phytools*^68^ v.1.9-16. Character history was inferred by stochastic character mapping with 1000 replicates and sampling character histories from their posterior probability distribution for the IQ-TREE phylogenetic tree, including all recognized species within the Picornaviridae.

## Supplementary information

Supplementary Information

## Data Availability

Genome sequences generated in this study have been deposited in NCBI GenBank under the accession numbers OR416237-OR416239.
