# Supplementary material for: Senecavirus cetus a novel picornavirus isolated from cetaceans represents a major host switching to the marine environment
Source: Npj Viruses. 2024 Aug 2;2:33. doi: 10.1038/s44298-024-00040-6 (PMC11721122; doi:10.1038/s44298-024-00040-6)
Supplement: Supplementary file 1 — Supplementary Information [file 44298_2024_40_MOESM1_ESM.pdf]

Supplemental Data

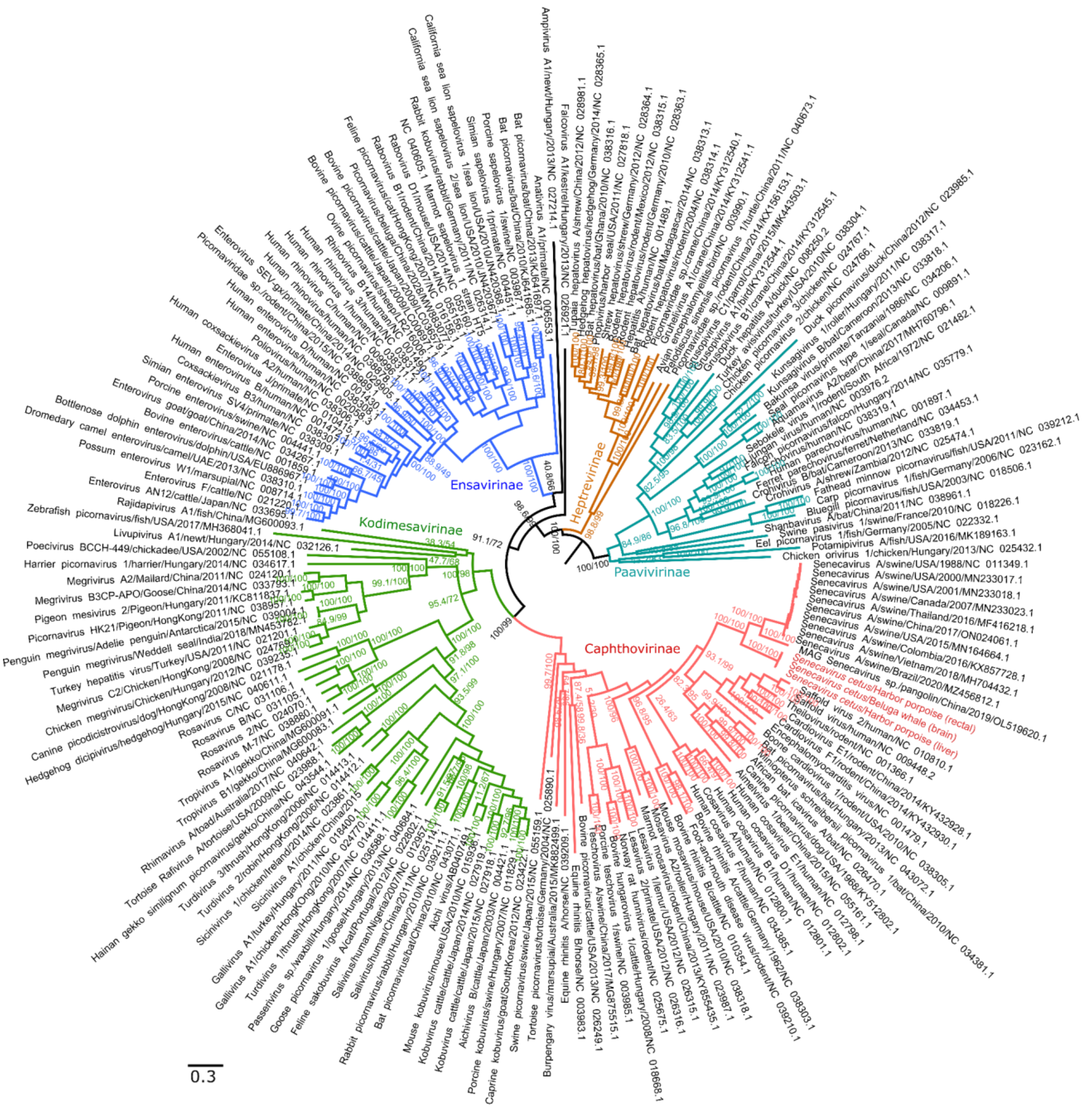

Supplemental Figure 1. Phylogenetic tree of the family Picornaviridae. Maximum likelihood phylogenetic tree was constructed in IQ-TREE v.1.6.12. Numbers at the nodes represent support values, approximate likelihood ratio test (aLRT)/ultrafast bootstrap (UFboot). Branches of the tree are coloured according to the four subfamilies within the Picornaviridae. The three isolates of the novel picornavirus species, *Senecavirus cetus*, are shown in red.

Supplemental table 1. Accession numbers and species names of picornavirus species used in the phylogenetic analysis.

Accession number	Species
NC_023985.1	*Aalivirus A*
AB040749.1	*Aichivirus A*
NC_015936.1	*Aichivirus A*
NC_004421.1	*Aichivirus B*
NC_023422.1	*Aichivirus C*
NC_011829.1	*Aichivirus C*
NC_027919.1	*Aichivirus D*
NC_027918.1	*Aichivirus D*
NC_039211.1	*Aichivirus E*
NC_043071.1	*Aichivirus F*
NC_055161.1	*Ailurivirus A*
NC_027214.1	*Ampivirus A*
NC_006553.1	*Anativirus A*
MH760796.1	*Aquamavirus A*
NC_009891.1	*Aquamavirus A*
NC_008250.2	*Avihepatovirus A*
NC_038304.1	*Avisivirus A*
NC_024766.1	*Avisivirus B*
NC_024767.1	*Avisivirus C*
LC006971.1	*Boosepivirus A*
LC036579.1	*Boosepivirus B*
LR216006.1	*Boosepivirus C*
NC_026249.1	*Bopivirus A*
EU886967.1	Bottlenose dolphin enterovirus
NC_038303.1	*Bovine rhinitis A virus*
NC_010354.1	*Bovine rhinitis B virus*
NC_021178.1	*Cadicivirus A*
NC_040611.1	*Cadicivirus B*
JN420368.1	California sea lion sapelovirus 1
JN420367.1	California sea lion sapelovirus 2
NC_001479.1_	*Cardiovirus A*
NC_001366.1	*Cardiovirus B*
NC_038305.1	*Cardiovirus C*
NC_010810.1	*Cardiovirus D*
NC_009448.2	*Cardiovirus D*
KY432928.1	*Cardiovirus E*
KY432930.1	*Cardiovirus F*
NC_012800.1	*Cosavirus A*
NC_012801.1	*Cosavirus B*
NC_012802.1	*Cosavirus D*
NC_012798.1	*Cosavirus E*

NC_034385.1	*Cosavirus F*
KY312540.1	*Crahelivirus A*
NC_025474.1	*Crohivirus A*
NC_033819.1	*Crohivirus B*
MH368041.1	*Danipivirus A*
KJ641685.1	*Diresapivirus A*
KJ641697.1	*Diresapivirus B*
NC_038306.1	*Enterovirus A*
NC_038307.1	*Enterovirus B*
NC_001472.1	*Enterovirus B*
NC_002058.3	*Enterovirus C*
NC_001430.1	*Enterovirus D*
NC_038308.1	*Enterovirus D*
NC_001859.1	*Enterovirus E*
NC_033695.1	*Enterovirus F*
NC_021220.1	*Enterovirus F*
NC_008714.1	*Enterovirus F*
NC_034267.1	*Enterovirus G*
NC_004441.1	*Enterovirus G*
NC_038309.1	*Enterovirus H*
NC_038310.1	*Enterovirus I*
NC_010415.1	*Enterovirus J*
NC_038989.1	*Enterovirus K*
NC_029905.1	*Enterovirus L*
NC_039209.1	*Equine rhinitis A virus*
NC_003983.1	*Erbovirus A*
NC_016156.1	*Felipivirus A*
NC_039210.1	*Foot-and-mouth disease virus*
NC_024770.1	*Gallivirus A*
NC_018400.1	*Gallivirus A*
KY312541.1	*Gruhelivirus A*
KY312544.1	*Grusopivirus A*
KY312545.1	*Grusopivirus B*
MK443503.1	*Grusopivirus C*
NC_026921.1	*Harkavirus A*
NC_001489.1	*Hepatovirus A*
NC_027818.1	*Hepatovirus B*
NC_038313.1	*Hepatovirus C*
NC_028363.1	*Hepatovirus D*
NC_038314.1	*Hepatovirus E*
NC_038315.1	*Hepatovirus F*
NC_038316.1	*Hepatovirus G*
NC_028365.1	*Hepatovirus H*
NC_028981.1	*Hepatovirus H*

NC_028364.1	*Hepatovirus I*
NC_018668.1	*Hunnivirus A*
NC_025675.1	*Hunnivirus A*
NC_038317.1	*Kunsagivirus A*
NC_033818.1	*Kunsagivirus B*
NC_034206.1	*Kunsagivirus C*
NC_018506.1	*Limnipivirus A*
NC_023162.1	*Limnipivirus B*
NC_039212.1	*Limnipivirus C*
NC_032126.1	*Livupivirus A*
NC_040684.1	*Ludopivirus A*
OL519620.1	MAG: Senecavirus sp. isolate CPSV
NC_026315.1	*Malagasivirus A*
NC_026316.1	*Malagasivirus B*
MK882499.1	*Marsupivirus A*
NC_024120.1	*Megrivirus A*
NC_033793.1	*Megrivirus A*
NC_038957.1	*Megrivirus B*
KC811837.1	*Megrivirus B*
NC_039235.1	*Megrivirus C*
NC_024769.1	*Megrivirus C*
NC_021201.1	*Megrivirus C*
NC_034617.1	*Megrivirus D*
NC_039004.1	*Megrivirus E*
NC_034381.1	*Mischivirus A*
NC_043072.1	*Mischivirus B*
NC_026470.1	*Mischivirus C*
KY512802.1	*Mischivirus D*
NC_023987.1	*Mosavirus A*
NC_038318.1	*Mosavirus A*
KY855435.1	*Mosavirus B*
NC_025432.1	*Orivirus A*
NC_014412.1	*Oscivirus A*
NC_014413.1	*Oscivirus A*
NC_038319.1	*Parechovirus A*
NC_001897.1	*Parechovirus A*
NC_003976.2	*Parechovirus B*
NC_021482.1	*Parechovirus C*
NC_034453.1	*Parechovirus D*
NC_035779.1	*Parechovirus E*
NC_018226.1	*Pasivirus A*
NC_014411.1	*Passerivirus A*
NC_036588.1	*Passerivirus B*
MN453782.1	Penguin megrivirus isolate Weddell seal

MW883077.1	Picornavirus HMU-1
NC_055108.1	*Poecivirus A*
NC_022332.1	*Potamipivirus A*
MK189163.1	*Potamipivirus B*
NC_026314.1	*Rabovirus A*
NC_055156.1	*Rabovirus B*
NC_040605.1	*Rabovirus C*
NC_055160.1	*Rabovirus D*
NC_023988.1	*Rafivirus A*
NC_043544.1	*Rafivirus B*
NC_040642.1	*Rafivirus C*
MG600093.1	*Rajidapivirus A*
NC_038311.1	*Rhinovirus A*
NC_038312.1	*Rhinovirus B*
NC_001490.1	*Rhinovirus B*
NC_009996.1	*Rhinovirus C*
NC_038878.1	*Rhinovirus C*
KX156153.1	*Rohelivirus A*
NC_024070.1	*Rosavirus A*
NC_038880.1	*Rosavirus A*
NC_031105.1	*Rosavirus B*
NC_031106.1	*Rosavirus C*
NC_022802.1	*Sakobuvirus A*
NC_025114.1	*Salivirus A*
NC_012957.1	*Salivirus A*
NC_003987.1	*Sapelovirus A*
NC_004451.1	*Sapelovirus B*
MZ456812.1	*Senecavirus A*
MN233023.1	*Senecavirus A*
ON024061.1	*Senecavirus A*
KX857728.1	*Senecavirus A*
MF416218.1	*Senecavirus A*
NC_011349.1	*Senecavirus A*
MN233017.1	*Senecavirus A*
MN233018.1	*Senecavirus A*
MN164664.1	*Senecavirus A*
MH704432.1	*Senecavirus A*
NC_038961.1	*Shanbavirus A*
NC_023861.1	*Sicinivirus A*
NC_028380.1	*Sicinivirus A*
NC_003985.1	*Teschovirus A*
MG875515.1	*Teschovirus B*
NC_025890.1	*Torchivirus A*
NC_055159.1	*Tottorivirus A*

NC_003990.1	*Tremovirus A*
NC_040673.1	*Tremovirus B*
MG600091.1	*Tropivirus A*
MG600083.1	*Tropivirus B*
